# Self-reported and accelerometer-based assessment of physical activity in older adults: results from the Berlin Aging Study II

**DOI:** 10.1038/s41598-023-36924-5

**Published:** 2023-06-21

**Authors:** Valentin Max Vetter, Duygu Deniz Özince, Jörn Kiselev, Sandra Düzel, Ilja Demuth

**Affiliations:** 1 Corporate Member of Freie Universität Berlin and Humboldt-Universität zu Berlin, Department of Endocrinology and Metabolic Diseases (Including Division of Lipid Metabolism), Lipid Clinic at the Interdisciplinary Metabolism Center, Biology of Aging Working Group, Charité – Universitätsmedizin Berlin, Augustenburger Platz 1, 13353 Berlin, Germany; 2 Max-Planck Institut für Bildungsforschung, Berlin, Germany; 3 Corporate Member of Freie Universität Berlin and Humboldt-Universität zu Berlin, Department of Anesthesiology and Operative Intensive Care Medicine (CVK/CCM), Charité – Universitätsmedizin Berlin, Chariteplatz 1, 10117 Berlin, Germany; 4 Department of Cardiology, Charité – Universitätsmedizin Berlin (CBF), Berlin, Germany; 5 Berlin Institute of Health at Charité – Universitätsmedizin Berlin, BCRT - Berlin Institute of Health Center for Regenerative Therapies, Berlin, Germany

**Keywords:** Epidemiology, Geriatrics

## Abstract

Physical activity (PA) has a substantial impact on health and mortality. Besides questionnaires that rely on subjective assessment of activity levels, accelerometers can help to objectify an individual’s PA. In this study, variables estimating PA and sleep time obtained through the wGT3X-BT activity monitor (ActiGraph LLC, USA) in 797 participants of the Berlin Aging Study II (BASE-II) were analyzed. Self-reports of PA and sleep time were recorded with Rapid Assessment of Physical Activity (RAPA) and the Pittsburgh Sleep Quality Index sleep questionnaire (PSQI). Total cholesterol (TC), high density lipoprotein cholesterol (HDL-C), low density lipoprotein cholesterol (LDL-C), triglycerides (TG), fasting glucose, and hemoglobin A1c (HbA1c) were determined in an accredited standard laboratory. Of all participants, 760 fulfilled the PA wear-time criteria. In this sample mean age was 75.6 years (SD: 3.8 years, range 66.0–94.1 years) and 53% of the included participants were women. Average wear time was 23.2 h/day (SD 1.3 h/day). Statistically significant differences between RAPA groups were found for all accelerometric variables except energy expenditure. Post-hoc analysis, however, suggested low agreement between subjective and device-based assessment of physical activity. TC, HDL-C, LDL-C, TG, fasting glucose and HbA1c were weakly correlated with accelerometric variables (Pearson’s r ≤ 0.25). Device-based average sleep time per night (mean sleep time = 6.91 h, SD = 1.3, n = 720) and self-reported average sleep time per night (mean sleep time = 7.1 h, SD = 1.15 h, n = 410) were in a comparable range and moderately correlated (Pearson’s r = 0.31, *p* < 0.001, n = 410). Results from this study suggest that self-reported PA obtained through the RAPA and device-based measures assessed by accelerometers are partially inconsistent in terms of the physical activity level of the participants. Self-reported and device-based measures of average sleep time per night, however, were comparable.

## Introduction

The impact of physical activity (PA) on health and mortality is undisputed^1,2^. Inactivity was estimated to be responsible for 7.2% of premature deaths and between 1.6% and 8.1% of non-communicable diseases like hypertension, type 2 diabetes, coronary heart disease, dementia and several forms of cancer (reviewed in^3^). The extent and prevalence of inactivity varies around the world, but on average, one-third of all adults do not achieve the PA levels recommended in public health guidelines^4^. To account for and examine activity related effects, a reliable assessment of PA on a daily basis is pivotal in epidemiological health studies. However, PA is a multidimensional process^5^ and to date no gold standard for its assessment is available^6^. Questionnaires on PA impress with their simplicity, low costs, and easy repeatability but were shown to have limited validity when compared to other methods^6^ and differ in the degree of resolution and time-frame of assessing PA. Another way of measuring PA is through accelerometers that assess more objectively the acceleration of the body part they are attached to (often in three axes). Decreasing costs, ease of use, and low invasiveness have made them an often employed tool even in larger cohorts^7^. In addition to providing information about participants' PA levels, accelerometers often allow estimation of sleep duration (and sometimes sleep quality), further increasing the value of this data collection method. However, accelerometers produce an immense volume of data and different approaches to reduce it to one single metric (which would be needed for easy use in most subsequent analyses) are available^8^. Still, due to the complexity and multidimensionality of PA^5^ there is no clear consensus which of these metrics would be best to measure overall activity^8–10^. Device-based and subjective measures were shown to differ^11,12^ and it was suggested that both measures should be used side by side^13,14^. Beyond that, it was shown that device-based measures differ in dependence on the body position where the monitor was worn^10,15–17^, employed filters^18^, data processing procedures and used algorithms for the aggregation of raw data (reviewed in^9^).

The complexity of PA and its measurement as described above are challenges that need to be addressed when using respective measures, be it as the focus of an analysis or as a confounder. To do so for the Berlin Aging Study II (BASE-II), a interdisciplinary longitudinal study of more than 2,000 participants at baseline^19,20^, we analyzed all available device-based and self-reported measures of PA and sleep from the follow-up which was part of the GendAge study. The primary objective of this manuscript is the detailed description of multiple variables measured with the wGT3X-BT monitor in a sample of 797 older adults with a mean age of 75.6 years (SD: 3.8 years). In a second step we aimed to compare device-based measurements with self-reported PA and sleep time. Subsequently and to evaluate the device-based PA variables even further, we analyzed them in relation to known PA-associated variables like age, Body Mass Index (BMI) as well as laboratory parameters. The analyses reported in this study will allow a better evaluation of the informative value of the regarding variables in the BASE-II cohort and enable future analyses to make use of these important measures.

## Methods

### Participants

A total of 1,100 participants originating from the Berlin Aging Study II (BASE-II)^19^ with an age of ≥ 60 years at baseline (52.1% women) were re-invited and medically assessed as part of the GendAge study. The time interval between baseline examination and follow-up was on average 7.4 years^20^. In this study we analyzed data that was collected during the follow-up examination as part of the GendAge study, which was completed between 2018 and 2020^20^.

### Actigraphy raw data assessment and data processing with the Actilife software package

BASE-II participants were asked to wear the wGT3X-BT activity monitor (ActiGraph LLC, USA) like a wristwatch (i.e. usually their non-dominant wrist) for the whole period between the second and the third day of examination at follow-up which were on average six days apart (SD = 0.85 days, range: 2 to 13 days). The wrist placement was chosen to increase the participants motivation and compliance to wear the device as long as possible. Additionally, they were provided with instructions on how to wear the accelerometer correctly and retrieved a summary report after they returned the accelerometers to the study site. Actigraph’s accelerometers are under the most used devices to analyze older cohorts and were used as reference standard for the evaluation of other activity monitors^21^.

All devices were initiated before they were assigned to each participant according to the manufacturer’s instructions (default settings).The manufacturers software package ActiLife 6 (ActiGraph LLC, USA) was used for data processing. Raw data was downloaded and aggregated into 10 s epochs. The low-frequency extension filter that increases the devices sensitivity to low-force accelerations was not applied to the data in this study^18^. Following the recommendations of Keadle and colleagues^22^ and Choi and colleagues^23^ the wear time validation algorithm by Choi implemented in the ActiLife software package was used to determine wear time of all participants. The algorithm derived wear time information was supplemented by the built-in wear time sensor of the wGT3X-BT monitor. Arguello and colleagues^24^ showed that the wear sensor has a lower sensitivity but a higher specificity in wear time detection than the implemented wear time validation algorithms. Therefore, conflicts between the Choi algorithm and the wear time sensor were manually solved following predefined rules:

- If the wear time sensor indicated non-wear time and algorithm indicated wear time, non-wear time was chosen.
- If wear sensor indicated wear-time and algorithm indicated non-wear time, non-wear time was chosen.

Periods of conflict shorter than 30 min were ignored, as their influence on the overall wear time estimation would be limited.

Biometric information such as age, sex, weight, and height were entered manually for each participant. Energy expenditure was calculated based on the algorithm by Freedson and colleagues^25^ (“Freedson VM3 (2011)” option in ActiLife 6) and cut points for intensity of PA were defined by Keadle and colleagues^22^ (“Keadle Women’s Health VM (2014)” option in ActiLife 6). Because most algorithms were developed from data that was derived through waist-worn devices, a correction of the wrist-derived data is necessary. This can be done by the “worn on wrist” option within the ActiLife 6 software package which is based on Actigraphs internal research and development (https://actigraphcorp.my.site.com/support/s/article/What-does-the-Worn-on-Wrist-option-do-in-the-Scoring-tab). According to personal communication with the manufacturer, this correction does not affect the step count calculation. The algorithm used to measure step counts is proprietary information and therefore not publicly available.

### Actigraphy data cleaning: activity variables

Data cleaning of the processed actigraphy data was done with the R software package, version 3.6.2^26^. The first and last day of each individual’s wear time was excluded, because it did not contain information for the whole day and presumably was heavily confounded by measurements that were recorded during the time the device was delivered to or from the study site. Daily wear time was calculated by adding up the wear time periods for each individual day. Average daily wear time was calculated by dividing the cumulative wear time (over the whole wear time period) by the number of days the monitor was worn. Only participants who wore the device for 5 days or more with an average daily wear time of 15 h or more were included in the statistical analyses presented in this study resulting in a mean wear time of the final sample of 6.055 days (SD = 0.55 days, range: 5 to 11 days).

### Actigraphy data cleaning: sleep variables

Data on sleep time was available for 784 participants. We excluded 39 participants because they did not fulfill our pre-defined wear time requirements. One participant was excluded because he provided data on less than three sleep time periods. Time in bed and sleep time were extracted from data obtained through the wGT3X-BT monitor by the algorithms implemented in the ActiLife 6 software package. The monitor was handed out and collected directly at the study center. Each participants sleep time periods were inspected individually for plausibility by the same investigator and obvious misclassifications were corrected manually. After manual correction almost no sleep periods over the day remained.

The average sleep time at night was calculated by dividing the sum of all recorded sleep time periods by the number of sleep time periods. If the number of sleep time periods exceeded the number of nights (assessed as number of days the monitor was worn plus one), the discrepancy needs to be attributed to longer wake periods during the night or to the very rarely occurring sleep time during the day (in addition to sleep time at night). In these cases, the average sleep time per night was calculated as follows: av. sleep time = sum of all sleep time periods/number of days monitor was worn + 1. In contrast to the days that were analyzed with respect to the activity variables (first and last day excluded), all nights between the day the participant received the device and the day they handed it over at the study site can be analyzed because the participants (if applicable) wore the device before they went to bed on the first day and until after they got out of bed on the last day and therefore for complete nights only. Furthermore, no interference due to transportation of the device needs to be accounted for during the sleep period. Consequently, the available nights exceed the available days by one.

A subgroup of participants that partly overlaps with participants that wore the wGT3X-BT monitor filled in the Pittsburgh Sleep Quality Index sleep questionnaire (PSQI). Participants whose difference between the device-based (wGT3X-BT monitor) and self-reported (PSQI questionnaire) sleep time exceeded 50% of the self-reported sleep time, were excluded, because a measurement error seems to be very likely (n = 24). After exclusion, sleep time variables obtained from the wGT3X-BT monitor and ActiLife 6 were available for 720 participants. A subgroup of 410 participants provided additionally self-reported information about the sleep time.

### Rapid assessment of physical activity (questionnaire)

Self-administered PA was assessed via the Rapid Assessment of Physical Activity (RAPA), a questionnaire that was developed to measure PA in older adults by Topolski and colleagues^27^ and analyzed in the BASE-II cohort before^28–30^. RAPA consists of seven questions about frequency and intensity of PA throughout the week. It is scored by choosing the highest ranked affirmed question which leads to categorization of PA into sedentary (1), “under-active” (2), “regular under-active (light activities)” (3), “regular under-active” (4 and 5), and “regular active” (6 and 7). At the time of assessment no validated German version was available but a very recent preprint publication reports on the validation of the RAPA in a cohort of 13 Austrian German speaking adults between 55 and 78 years of age .

### Clinical phenotypes and blood parameters

Height and weight were measured with the electronic measuring station “seca 763” (SECA, Germany). All blood parameters were measured in a standard laboratory. Cholesterol, triglycerides, high density lipoprotein cholesterol (HDL-C) and low-density lipoprotein cholesterol (LDL-C) were determined by enzymatic colorimetry. Serum fasting glucose was measured using the UV test. A turbidimetric inhibition immunoassay (TINIA) was employed to quantify glycated hemoglobin A1c.

### Imputation of missing values in actigraphy variables

To be able to make use of the accelerometric variables as covariates in future analyses that may e.g. include the complete number of participants (n = 1100), missing values were imputed. The substitution of missings by the variable mean attenuates possible correlations to other variables, which is known to cause problems in multivariable analyses for which we want to use it in the future. Hence, we chose to employ stochastic regression imputation, which, in contrast to deterministic regression imputation, includes a random error term to predict the value of the missing data points based on variables known for all participants (chronological age, sex, BMI). Results of imputation were manually checked for plausibility. Of the 1,100 participants that were included in the analyzed cohort, 1,098 participants provided information about their BMI, age and sex. To be able to include as many participants as possible for the imputation, less strict inclusion criteria were employed. In concordance with previous analyses^31^, participants who provided data on two days or more with an average wear time of ten hours or more were included. Missing values for 306 participants (27.9%) were imputed by stochastic regression imputation with R’s “mice” package^32^.

### Statistical analyses

The statistical tests were done with the R software package, version 3.6.2^26^. Figures were produced with R’s “ggplot2” package^33^. A *p*-value < 0.05 was used to define statistical significance. Difference between group’s mean was assessed with t-tests and analyses of variance (ANOVA). The correlation plot was drawn with the *ggcorr* function of the “ggplot2” extension “GGally”. We performed available cases analyses; therefore, participants were only excluded from an analysis if they did not provide data on one of the variables included in the analysis. The number of observations is stated for every analysis individually. Please note that the imputed values described above were only used for analyses shown in Supplementary Table 2.

### Ethics approval and consent to participate

All participants gave written informed consent. All medical assessments were conducted in accordance with the Declaration of Helsinki and the study was approved by the Ethics Committee of the Charité – Universitätsmedizin Berlin (Approval Number EA2/144/16) and was registered in the German Clinical Trials Registry as DRKS00016157.

## Results

### Cohort characteristics and wear time

**Figure 1:**
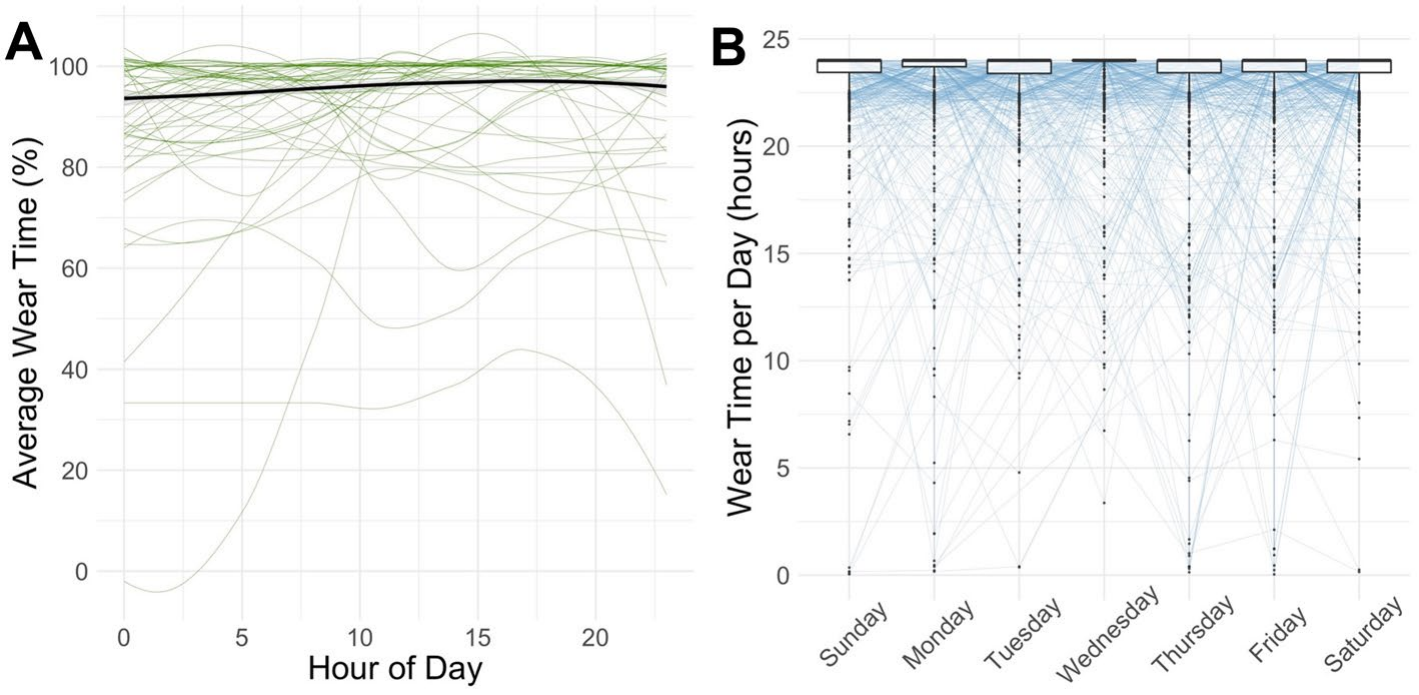
Analyses of average wear time (**A**) as a function of hour of the day and boxplots of average daily wear time (**B**) stratified by day of the week. Consistently high wear time was present through the day and the week. Median, hinges (25th and 75th percentile) and Tukey-style whiskers (1.5* inter-quartile-range) are displayed in the boxplots. Boxplot outliers were defined as values more extreme than 1.5*IQR.

Descriptive statistics are displayed in Table 1. Of all 797 participants who wore the accelerometric monitor, 760 fulfilled our wear-time based inclusion criteria. Participants had a mean age of 75.6 years (SD: 3.8 years, range: 66.0–94.1 years) and 53% of the analyzed sample were women. Participants wore the device for on average six days for on average 23.2 h (SD: 1.3 h) per day (Table 1). To identify possible under- or overestimation of total PA due to potential wear time preferences over the day, the week, or months of the year, stratified wear time validation analyses were conducted and no systematic irregularities in wear time were found (Fig. 1 and Supplementary Fig. 1). Furthermore, no variance in wear time with respect to the number of days the device was already worn was found (data not shown).

**Table 1:** Cohort characteristics of BASE-II participants that provided accelerometric data obtained through ActiGraph’s wGT3X-BT monitor.

Variables		n	Mean, %	SD	Min	Max
Age (years)		760	75.61	3.81	65.95	94.07
Sex	Men	357	46.97			
	Women	403	53.03			
RAPA Score	1	2	0.26			
	2	4	0.53			
	3	103	13.57			
	4	272	35.84			
	5	91	11.99			
	6	226	29.78			
	7	61	8.04			
Seldom/never physically active^1^	Yes	96	12.65			
	No	663	87.35			
Vector magnitude (counts × 1000)		760	19,310.00	5,481.00	4,604.00	43,830.00
Step count		760	12,050.00	3,415.00	2,664.00	26,070.00
Energy expenditure (kcal)		760	1540.00	574.40	45.72	4,734.00
MET (average per hour)		760	1.42	0.16	1.03	2.01
Physical activity: sedentary (hours/day) [%/hour]^a^		760	11.24 [49]	1.88 [7]	3.24 [26]	17.44 [73]
Physical activity: light (hours/day) [%/hour]^a^		760	6.56 [29]	1.20 [5]	1.88 [14]	10.56 [49]
Physical activity: moderate (hours/day) [%/hour]^a^		760	5.04 [22]	1.60 [7]	0.36 [2]	10.12 [47]
Average wear time (hours/day)		760	23.15	1.31	15.59	24.00
Sleep time (hours/night), wGT3X-BT monitor		720	6.91	1.25	3.27	11.49
Sleep time (hours/night), questionnaire		410	7.07	1.15	4	11
Difference sleep time (wGT3X-BT—questionnaire)		410	− 0.08	1.37	− 4.45	3.32

^1^According to RAPA item 1.

^a^Please note that the hours per day spent on each activity category do not sum up to the full 24 h, as the average time per day the device was not worn must be included. The percentage value per hour indicates the share of the respective activity category in all activities recorded per hour (and therefore sums up to 100% as the time during which the device was not worn is not included in the calculation). In addition, the Actilife software package evaluates wear time separately from PA variables by algorithm and wear time sensor, as described in the Methods. Therefore, differences between the sum of PA per day and the average wear time per day are to be expected.

*SD* standard deviation, min: minimum, *max* maximum, *RAPA* Rapid Assessment of Physical Activity, *kcal* kilocalorie, *MET* Metabolic Equivalent of Task.

### Physical activity

Descriptive statistics of the PA variables that were derived from the wGT3X-BT monitor are shown in Table 1. We found higher PA in men when assessed as Vector Magnitude, Step Count, Energy Expenditure and cut-off defined PA (sedentary, light, and moderate, *p* ≤ 0.002, Supplementary Table 1).

**Figure 2:**
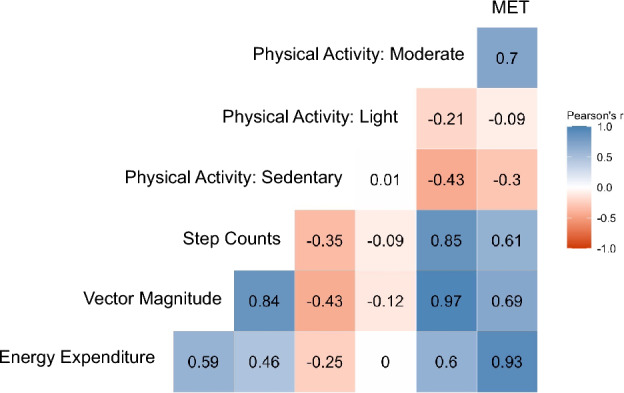
Heatmap of Pearson’s correlation between the available accelerometric variables. Correlation coefficients were calculated as Pearson’s r. *Note MET* Metabolic Equivalent of Task.

A moderate to high positive correlation was found between Vector Magnitude, Step Counts, Energy Expenditure, time spent in moderate PA and Metabolic Equivalent of Task (MET, Pearson’s r between 0.5 and 0.97, Fig. 2).

As expected, the two variables indicating lower PA (sedentary and light PA) were negatively associated with the other PA variables (Pearson’s r between 0 and − 0.4, Fig. 2).

Normalized values of the different activity variables over the course of the day are depicted in Supplementary Fig. 2. We found only moderate seasonal changes in activity over the course of the year (Supplementary Fig. 3) with the highest amount of PA around April. The differences are, however, rather small, and we would not expect them to impose considerable bias to our analyses.

### Comparison of device-based and subjective (self-rated) physical activity

**Figure 3:**
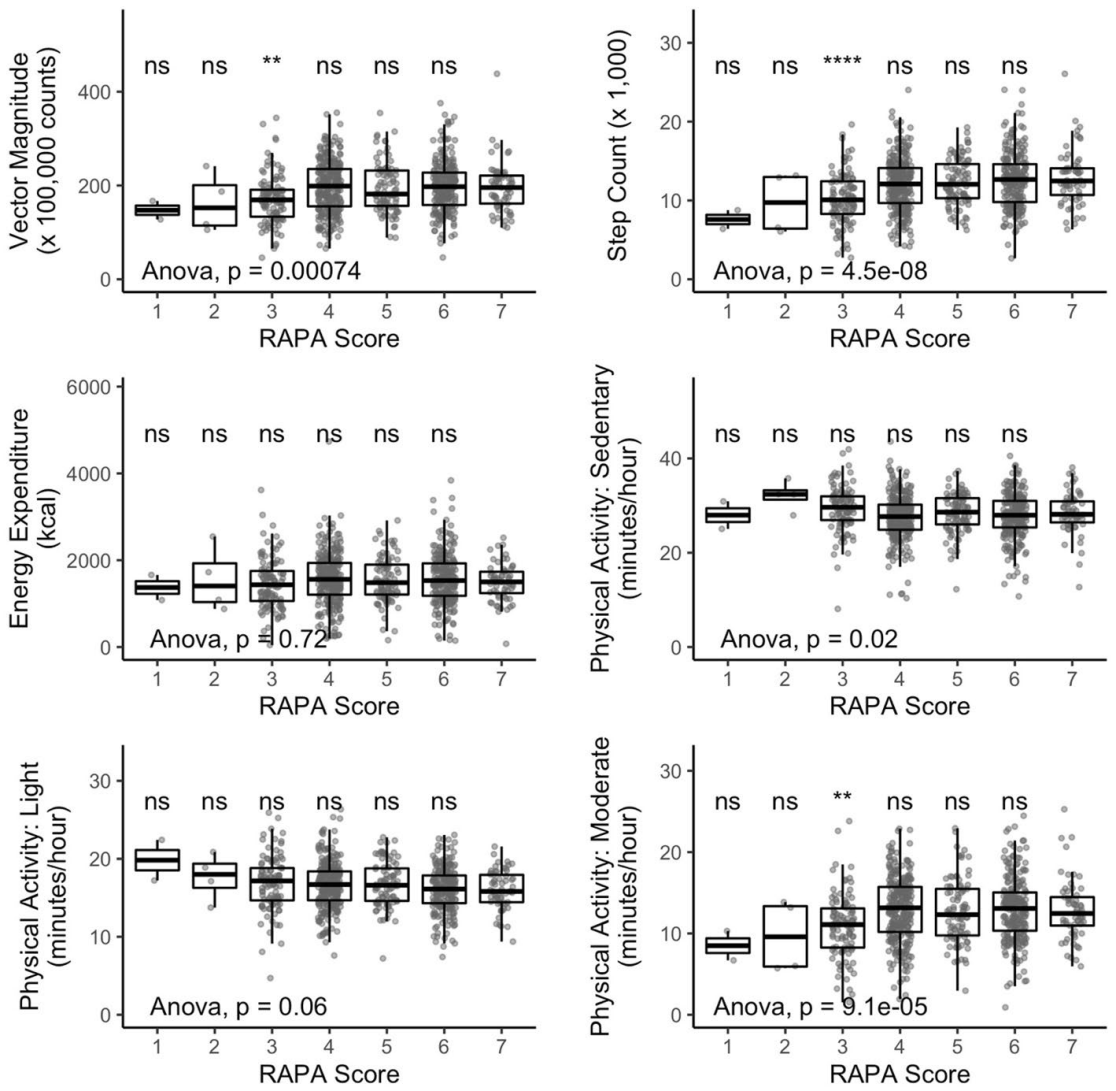
Results in activity variables stratified by self-rated physical activity (assessed via RAPA questionnaire). Statistical significance of difference between means was assessed by ANOVA. Difference between the highest self-rate activity (RAPA = 7) and the other RAPA groups were tested for statistical significance with *t* test. N = 759. In contrast to results in Table 1, Physical Activity is presented in minutes/hour. It can be transformed as follows: [minutes/hour] = ([hours/day]*60)/24. **p ≤ 0.01, ****p ≤ 0.0001.

To explore how device-based and subjective PA were linked in this cohort, we compared results obtained from the accelerometer with self-reported PA assessed with the well-established Rapid Assessment of Physical Activity (RAPA). The RAPA contains seven yes/no-questions and categorizes participants based on self-rated PA. We found statistically significant differences in all accelerometric PA indicators between the RAPA groups except for Energy Expenditure and average minutes per hour spent with light PA (*p* ≤ 0.02, ANOVA, Fig. 3).

However, when performing a post-hoc analysis comparing the highest PA group (RAPA group 7 = “regular active”) with the other RAPA groups, significant differences were only found in group 3 in Vector Magnitude, Step Count, and moderate PA (t-Test, Fig. 3). Furthermore, we found differences in accelerometric variables to be higher between RAPA groups indicating low self-rated PA (e.g. group 1 to 3, Fig. 3) compared to differences between RAPA groups indicating that the participants were “under-active” or “active” with respect to PA (groups 4 to 7). Sex-stratified analyses showed similar results and are displayed in Supplementary Fig. 4.

**Figure 4:**
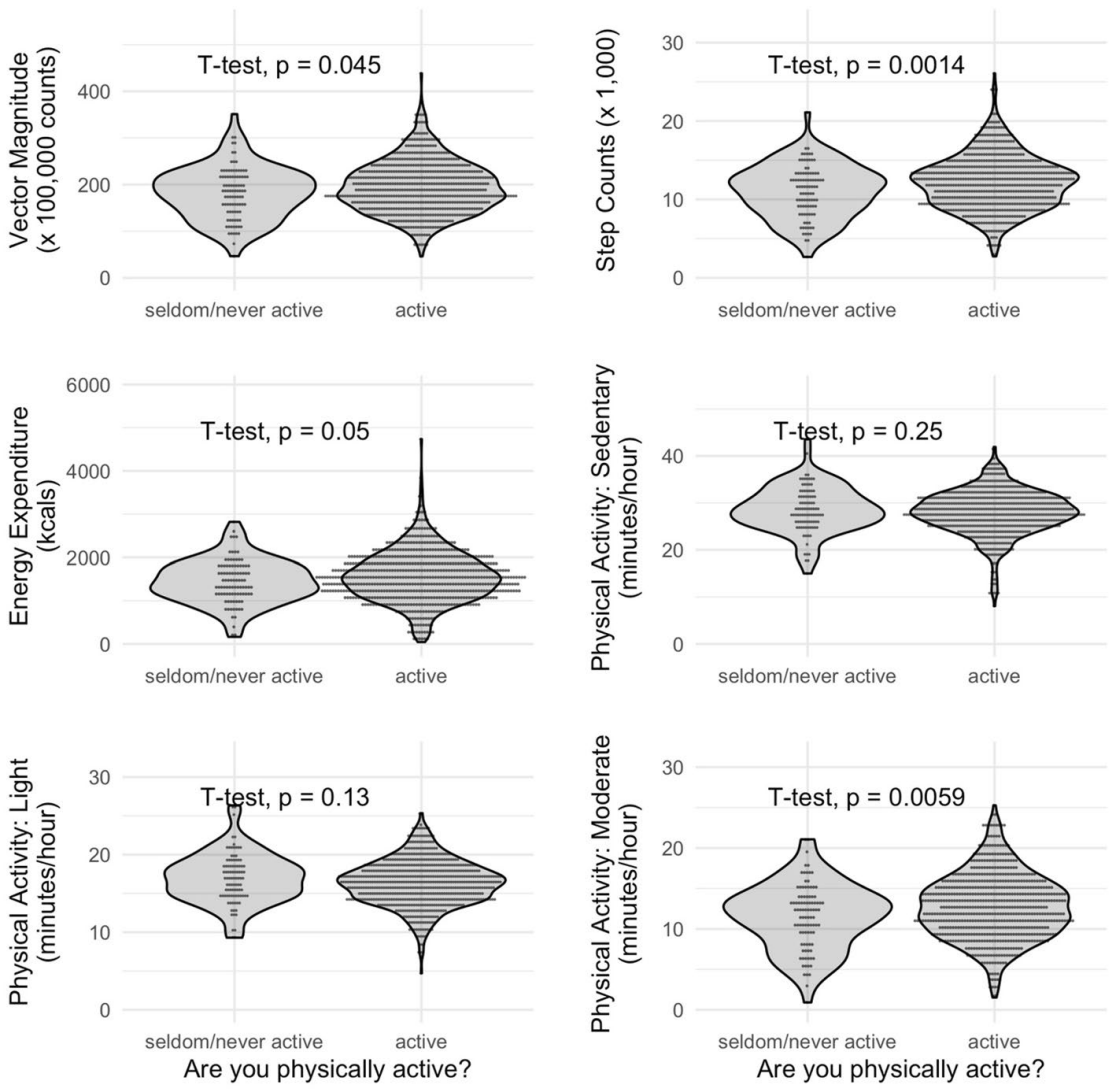
Violin plots of accelerometric activity variables of participants that reported to be seldom/never active or to be active. Statistical significance of difference between means was calculated using t-test. N = 759. Vector Magnitude (never active: mean = 182, active: mean = 195), Step Counts (never active: mean = 11, active: mean = 12), Energy Expenditure (never active: mean = 1443, active: mean = 1554), PA: sedentary (never active: mean = 29, active: mean = 28), PA: light (never active: mean = 17, active: mean = 16), PA: moderate (never active: mean = 12, active: mean = 13). In contrast to results in Table 1, Physical Activity is presented in minutes/hour. It can be transformed as follows: [minutes/hour] = ([hours/day]*60)/24.

Subsequently, we examined differences in the accelerometric variables between participants who rated themselves based on the first RAPA question to be “rarely/never active” or “active”. We found that participants who considered themselves as physically active showed higher PA levels measured as Vector Magnitude, step counts, and cut-off defined moderate PA compared to the more sedentary participants stating that they were seldom or never active (*p* < 0.05, t-Test, Fig. 4). In sex-stratified follow-up analyses, this association remained significant for step counts only in the female and male subgroup and for moderate PA in the female subgroup only (Supplementary Fig. 5).

### Relationship between physical activity, age, BMI, and blood parameters

We found a weak negative correlation between age and PA (Vector Magnitude, step count, energy expenditure, MET, and moderate PA; Pearson’s r between − 0.15 and − 0.18, n = 760, Table 2). No significant correlation was found between age and variables indicating sedentary or light PA (Pearson’s r < 0.1, Table 2). Interestingly, a moderate positive correlation was found between energy expenditure and BMI (Pearson’s r = 0.4), although step counts and vector magnitude showed a negative correlation with this variable (Pearson’s r = -0.2, n = 760, Table 2). This finding most likely results from the way energy expenditure is calculated by the Actilife software package which incorporates the individual’s weight. Weak correlations were found between activity variables and TC, LDL-C, TG, fasting glucose and HbA1c. The strongest correlation was found between HDL and vector magnitude, step count and cut-off defined moderate PA (Pearson’s r = between 0.21 and 0.25, n = 750, Table 2).

**Table 2:** Table of correlation between activity variables (Actigraph), chronological age, BMI and blood parameters. Correlation was calculated as Pearson’s r.

		Age^1^	BMI^1^	Total Cholesterol^1^	HDL-C^2^	LDL-C^1^	TG^1^	Glucose^3^	HbA1c^4^
Vector magnitude (counts)	r	− 0.16	− 0.16	0.14	0.25	0.08	− 0.09	− 0.15	− 0.07
	*p*	< 0.001	< 0.001	< 0.001	< 0.001	0.030	0.010	< 0.001	0.048
Step count	r	− 0.15	− 0.26	0.11	0.21	0.06	− 0.13	− 0.17	− 0.11
	*p*	< 0.001	< 0.001	0.004	< 0.001	0.074	< 0.001	< 0.001	0.002
Energy expenditure (kcal)	r	− 0.18	0.4	− 0.09	− 0.12	− 0.06	0.09	0.09	0.08
	*p*	< 0.001	< 0.001	0.015	0.001	0.075	0.017	0.017	0.024
MET	r	− 0.16	0.22	− 0.01	− 0.04	0	0.04	0.01	0.01
	*p*	< 0.001	< 0.001	0.770	0.226	0.987	0.282	0.831	0.749
Physical activity: sedentary	r	0.09	0.05	0	− 0.13	0.02	0.08	0.07	0.01
	*p*	0.017	0.197	0.952	0.001	0.538	0.033	0.055	0.793
Physical activity: light	r	0.05	0.15	− 0.08	− 0.16	− 0.05	0.07	0.10	0,1
	*p*	0.144	< 0.001	0.026	< 0.001	0.153	0.069	0.004	0.004
Physical activity: moderate	r	− 0.15	− 0.18	0.12	0.25	0.05	− 0.1	− 0.16	− 0.09
	*p*	< 0.001	< 0.001	0.001	< 0.001	0.134	0.004	< 0.001	0.013

^1^n = 760, ^2^n = 750, ^3^n = 758, ^4^n = 756.

*MET* Metabolic Equivalent of Task.

*BMI* Body Mass Index, *HDL-C* high density lipoprotein cholesterol, *LDL-C* low density lipoprotein cholesterol, *TG* triglycerides, *HbA1c* hemoglobin A1c, *kcal* kilocalorie, *MET* Metabolic Equivalent of Task.

### Imputation of incomplete cases

To achieve a complete dataset for future analyses, stochastic regression imputation was used to substitute missing values of 306 participants as a function of chronological age, sex, and BMI. To validate the resulting dataset, the correlation analyses described above were repeated in the extended dataset (Supplementary Table 2). As expected and due to the methodological approach chosen for the imputation, correlation with age and BMI in the extended dataset was very similar to the results found in original dataset that includes only the measured results. A very high degree of agreement in correlation between variables was found for the blood parameters as well, which further increased confidence the informative value of imputed variables (Supplementary Table 2).

### Sleep

Self-reported data about sleep time was available for a subgroup 410 participants of all participants who wore the accelerometric monitor. An average sleep time of 7.1 h (SD = 1.15 h, range: 4–11 h, n = 410, Table 1) was reported by the participants in the PSQI questionnaire. Very similar results were measured with the wGT3X-BT monitor (mean sleep time = 6.91 h, SD = 1.3 h, range: 3.3–11.5 h, n = 720, Table 1). The average difference between device-based and subjective sleep time was − 0.1 h (SD = 1.4, range: − 4.5–3.3 h, n = 410, Table 1).

Subjective and device-based sleep time were moderately correlated (Pearson’s r = 0.31, *p* > 0.001, n = 410, Supplementary Fig. 6A). This was also true for sex-stratified subgroup analyses (women: Pearson’s r = 0.34, *p* < 0.001 and men: Pearson’s r = 0.29, *p* < 0.001, Supplementary Fig. 6B).

## Discussion

In this study we compare device-based assessments of PA (n = 760) and sleep time (n = 720) with self-reported estimates from participants originating from the BASE-II study aged between 66 and 94 years (53.0% women). Wear time was found to be high and independent from the time of the day, weekday, month, or season. Expected correlations were found between device-based assessments of activity levels and self-reported PA, BMI and blood parameters known to be associated with PA. Additionally, sleep time per night measured by the *wGT3X-BT monitor* was comparable with self-assessed reports of sleep time.

### Step counts are overestimated by wGT3X-BT accelerometers but allow relative comparison of individuals within one sample

Step counts in this cohort were in a comparable range with results from cohorts with a similar age distribution and accelerometric data derived from wrist-worn accelerometers^10,16^. Mandigout and colleagues measured 11,060 steps per day in 22 participants (mean age (SD): 76.6 (4.7), 36% women) from a wrist worn device in contrast to 5,922 steps that were obtained from waist worn monitors in the same participants at the same time^16^. A similar degree of overestimation was found by Kamada and colleagues who examined 94 women (mean age (SD): 71.9 (6.0) years) and reported 10,107 (wrist worn device) and 5378 steps (waist worn device) for the same activity period. Both authors conclude that the step count variable by the wGT3X-BT monitor (respectively the ActiLife software) is vastly overestimated when obtained from wrist-worn monitors. Although participants in the current study wore the accelerometer on their wrist without any comparison to waist-worn monitors, it is highly likely that a comparable degree of overestimation can be expected in our data, as well. Systematic overestimation may be attributed to the underlying proprietary ActiLife algorithm which was trained on data obtained from waist worn devices^10^. Furthermore, a waist worn device is closer to the body’s center of mass and might therefore be able to better capture the whole body’s acceleration^16^. Nevertheless, many sitting or standing activities may involve movements of arms. Thus, a wrist mounted accelerometer may detect this kind of acceleration induced by movements of the arms only, even when the participant is not walking^16^. It was argued that this measurement “may, however, be a good indicator of a more global amount of PA”^16^. Additionally, it is important to mention that the step count variable obtained from wrist and waist worn monitors were correlated in a previous study^16^ and correlate when classifying participants’ activity in quintiles^10^. Similar results were reported for Vector Magnitude^10,15^. These findings suggest that although the activity variables obtained from wrist worn devices probably cannot be used as absolute values, they can be informative on differences in PA within the analyzed group and might provide an even more complete assessment of PA as the waist worn monitors because it is able to include upper limb activity.

### Agreement between device-bases PA measures and self-reported PA is limited

The RAPA score is a widely used instrument to assess PA in older adults^27^. The first item of this questionnaire (“I rarely or never do any physical activities: yes/no”) was used to construct the Fried frailty measure in the BASE-II study^34–39^ and employed as covariate in multiple BASE-II publications^20,40–42^ before. Although one of our main results states that the degree of agreement between device-based PA assessment and results from the RAPA questionnaire as a whole is limited (which was shown for other PA questionnaires before^11,12^), statistically significant correlations between device-based measures of PA with answers to the first RAPA question were found. This suggests that although device-based and subjective PA (when derived from the complete RAPA questionnaire) differ and potentially should be used complementary^13,14^, the first RAPA question might provide a valuable compromise between both methods. However, this obviously only applies if a more complete analysis of device-based and self-assessed PA measures is not possible and cannot replace comprehensive PA assessments.

### Device-based PA measures is correlated with age, BMI and blood parameters

Our findings show that device-based PA variables and BMI, age and blood parameters are correlated. Interestingly, a positive correlation between BMI and energy expenditure was found (Pearson’s r = 0.4). This inverse finding probably needs to be attributed to the underlying algorithm for energy expenditure, which incorporates body weight and acknowledges the higher amount of energy that would be needed to carry a larger body mass. Additionally, weak correlations were found between higher HDL-C and higher levels of activity measured as Vector Magnitude, step counts, and time spent with moderate PA (Pearson’s r between 0.21 and 0.25). Correlations between blood parameters, Vector Magnitude and step counts found in this study were consistent with previous results reported by Wolff-Hughes and colleagues for TC, LDL-C, HDL-C, TG, glucose, and HbA1c^43^, although the effect size of the association was difficult to compare due to methodological differences. Additionally, the direction of correlations was the same for the bout-defined measure of moderate PA with the only exception of TC and LDL-C^43^. Although the available data and study design obviously do not allow any statements about causality between both measures, the correlation increases confidence in the device-based PA measures. Analyzed variables are known risk factors for cardiovascular events as is low PA. The presented associations between those variables that show the expected direction of effect draws a conclusive picture with respect to cardiovascular risk and add to the positive evaluation of the informative value of the device-based PA measures.

### Strengths and limitations

We would like to point out several limitations to this study. First, due to the position the monitor was worn, we were not able to measure accurately the absolute number of steps taken. However, this phenomenon is well-known and does not hinder the use of this variable relatively within the analyzed group. Second, the measured data was assessed under every-day life conditions and no diary-based information on PA was available for this period. Therefore, we were not able to directly validate the device-based data with information acquired under laboratory conditions or through detailed records of activity. However, we were able to show that the accelerometric activity data showed expected correlations with age, BMI and blood parameters. Therefore we are confident that the measured data provide valuable information about the participant’s individual PA level. Third, only a subgroup of the total sample of 1,100 participants of the analyzed cohort provided accelerometric data. To be able to make use of the whole dataset in future analyses, the missing values were substituted by a stochastic regression imputation. Although the fraction of imputed values in the final dataset is large (27.9%), results of correlation analyses between the extended dataset and blood parameters make the values seem plausible and therefore potentially valuable for use in future analyses.

Strengths of this study are the large number of participants who provided accelerometric data. Additionally, the wear time of the accelerometers was very high and independent from time of day, day of week or season. Due to the extensive and rich dataset of BASE-II we were able to relate the objectively measured PA data as well as sleep time to subjectively reported PA levels and self-reported sleep time. Additionally, we examined the relationship between the accelerometric variables and fitness-associated blood parameters. The different PA variables derived from the accelerometer can be used to comprehensively examine different aspects of PA in future analyses with BASE-II data e.g. in the context of cardio-metabolic health.

## Conclusion

Device-based PA measure’s correlation with age, BMI and laboratory-based variables suggest high informative value of the variables derived from the wGT3X-BT monitor in the BASE-II dataset. Although agreement between device-based PA measures and results from the complete RAPA score is limited, the first RAPA question on its own is statistically significantly associated with device-based PA variables. This suggests that although subjective and device-based PA measures should be used in a complementary manner whenever possible, the first RAPA question could potentially be a valuable compromise when a more detailed assessment of PA is not possible.

## Supplementary Information

Supplementary Information.

## Data Availability

Due to concerns for participant privacy, data are available only upon reasonable request. Please contact Ludmila Müller, scientific coordinator, at lmueller@mpib-berlin.mpg.de, for additional information.
