# Supplementary material for: Self-reported and accelerometer-based assessment of physical activity in older adults: results from the Berlin Aging Study II
Source: Sci Rep. 2023 Jun 21;13:10047. doi: 10.1038/s41598-023-36924-5 (PMC10284919; doi:10.1038/s41598-023-36924-5)
Supplement: Supplementary file 1 — Supplementary Information. [file 41598_2023_36924_MOESM1_ESM.pdf]

**Self-Reported and Accelerometer-Based Assessment of Physical Activity in Older Adults: Results from the Berlin Aging Study II**

Valentin Max Vetter[1], Duygu Deniz Özince[2], Jörn Kiselev[3], Sandra Düzel[2,4], Ilja Demuth[1,5]

[1]Charité – Universitätsmedizin Berlin, corporate member of Freie Universität Berlin and Humboldt-Universität zu Berlin, Department of Endocrinology and Metabolic Diseases (including Division of Lipid Metabolism), Biology of Aging working group, Augustenburger Platz 1, 13353 Berlin, Germany

[2]Max-Planck Institut für Bildungsforschung

[3]Charité – Universitätsmedizin Berlin, corporate member of Freie Universität Berlin and Humboldt-Universität zu Berlin, Department of Anesthesiology and Operative Intensive Care Medicine (CVK/CCM), Chariteplatz 1, 10117 Berlin, Germany

[4]Charité – Universitätsmedizin Berlin(CBF), Department of Cardiology, Berlin, Germany;

[5]Berlin Institute of Health at Charité – Universitätsmedizin Berlin, BCRT - Berlin Institute of Health Center for Regenerative Therapies, Berlin, Germany

**Corresponding author:**

Ilja Demuth (Ph.D.)
Charité - Universitätsmedizin Berlin
Lipid Clinic at the Interdisciplinary Metabolism Center,
Biology of Aging Group
Augustenburger Platz 1
13353 Berlin
Email: ilja.demuth@charite.de
Phone: ++49 30 450 569 143
FAX: ++49 30 450 566 904

Supplementary Material:

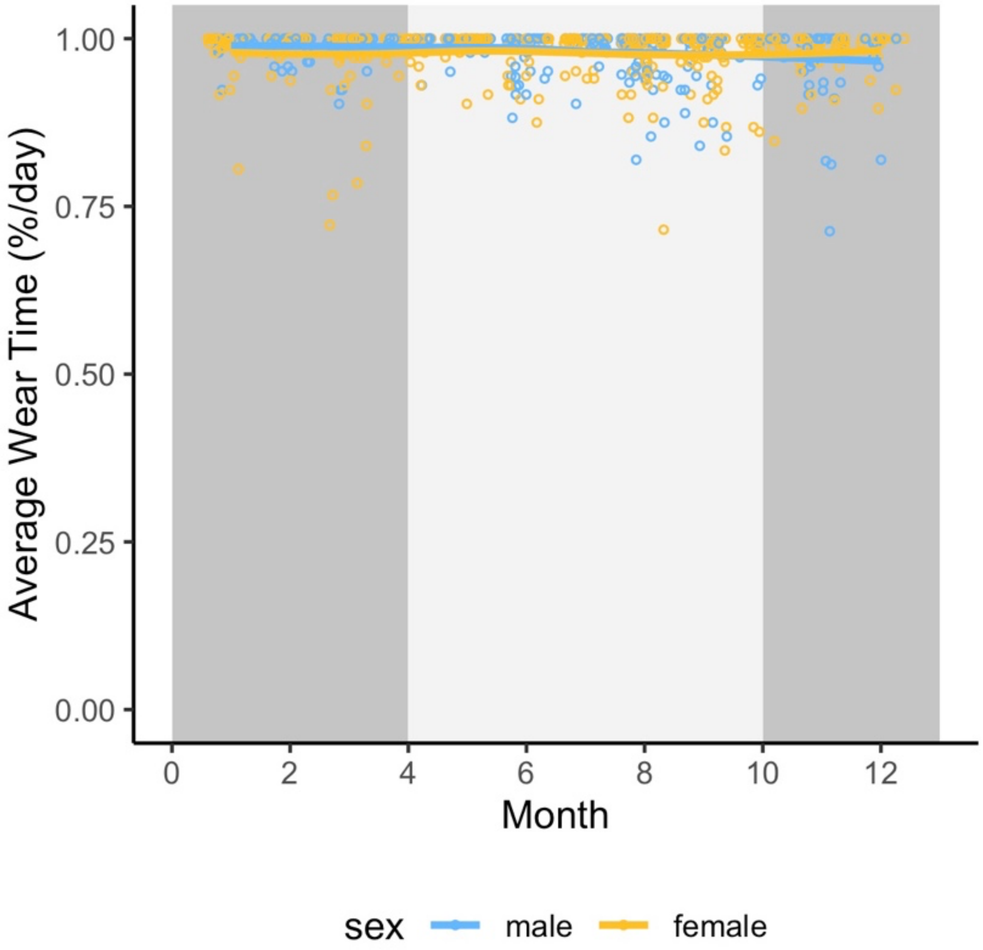


**Supplementary Figure 1: Average individual wear time of each participant per month of the first day of wear time.** Seasons are indicated by background color (dark grey = winter, light grey = summer).

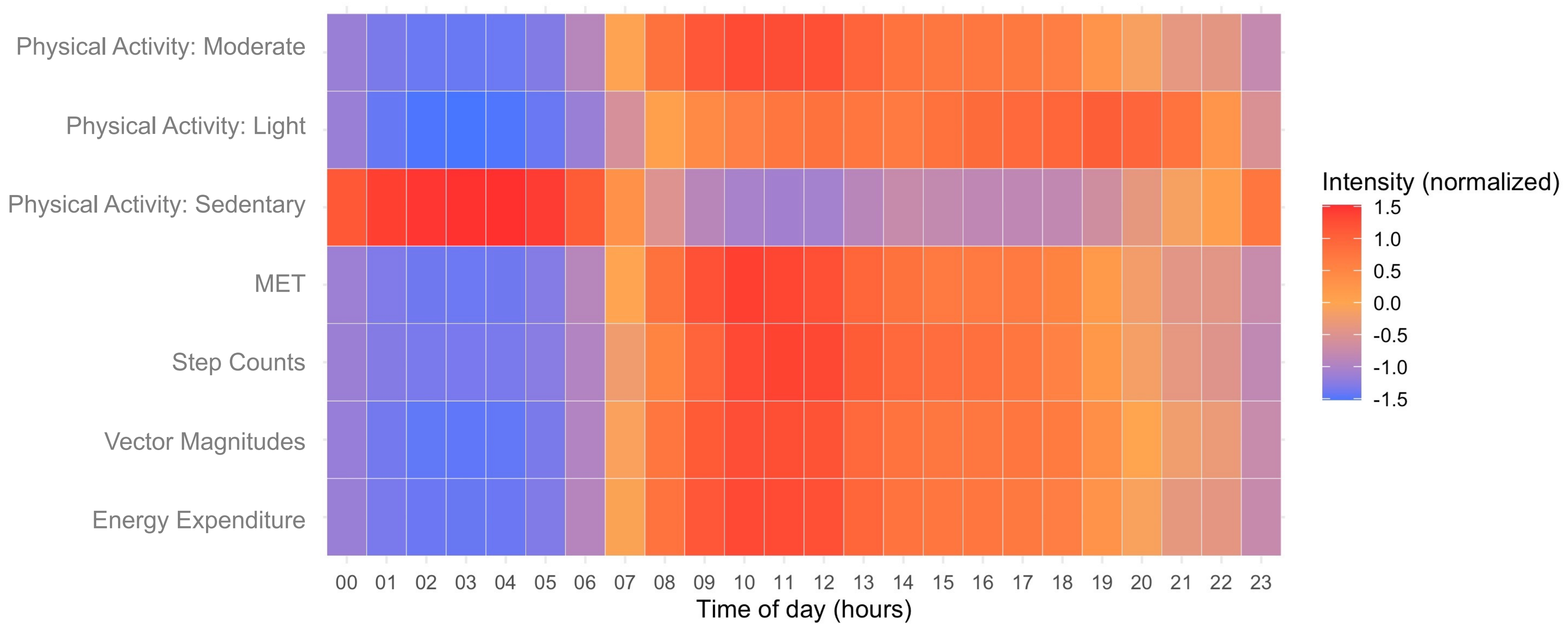


**Supplementary Figure 2: Heatmap of the normalized average values of the available activity variables over the course of the day.**

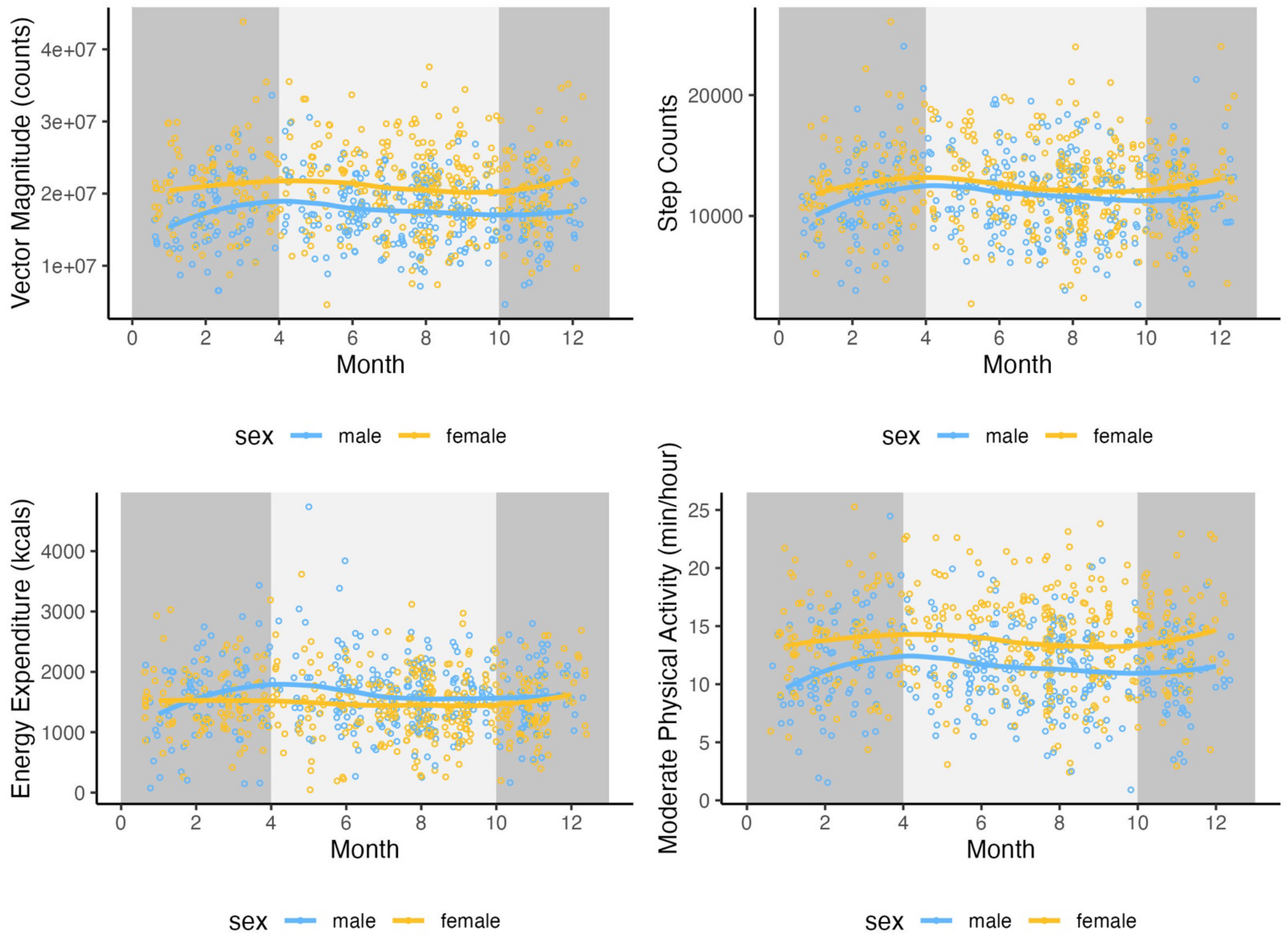


**Supplementary Figure 3: Average physical activity of BASE-II participants over the course of the year.** Season is indicated by background color (dark grey = winter, light grey = summer). In contrast to results in Supplementary Table 1, Physical Activity is presented in minutes/hour. It can be transformed as follows: [minutes/hour]= ([hours/day]*60)/24.

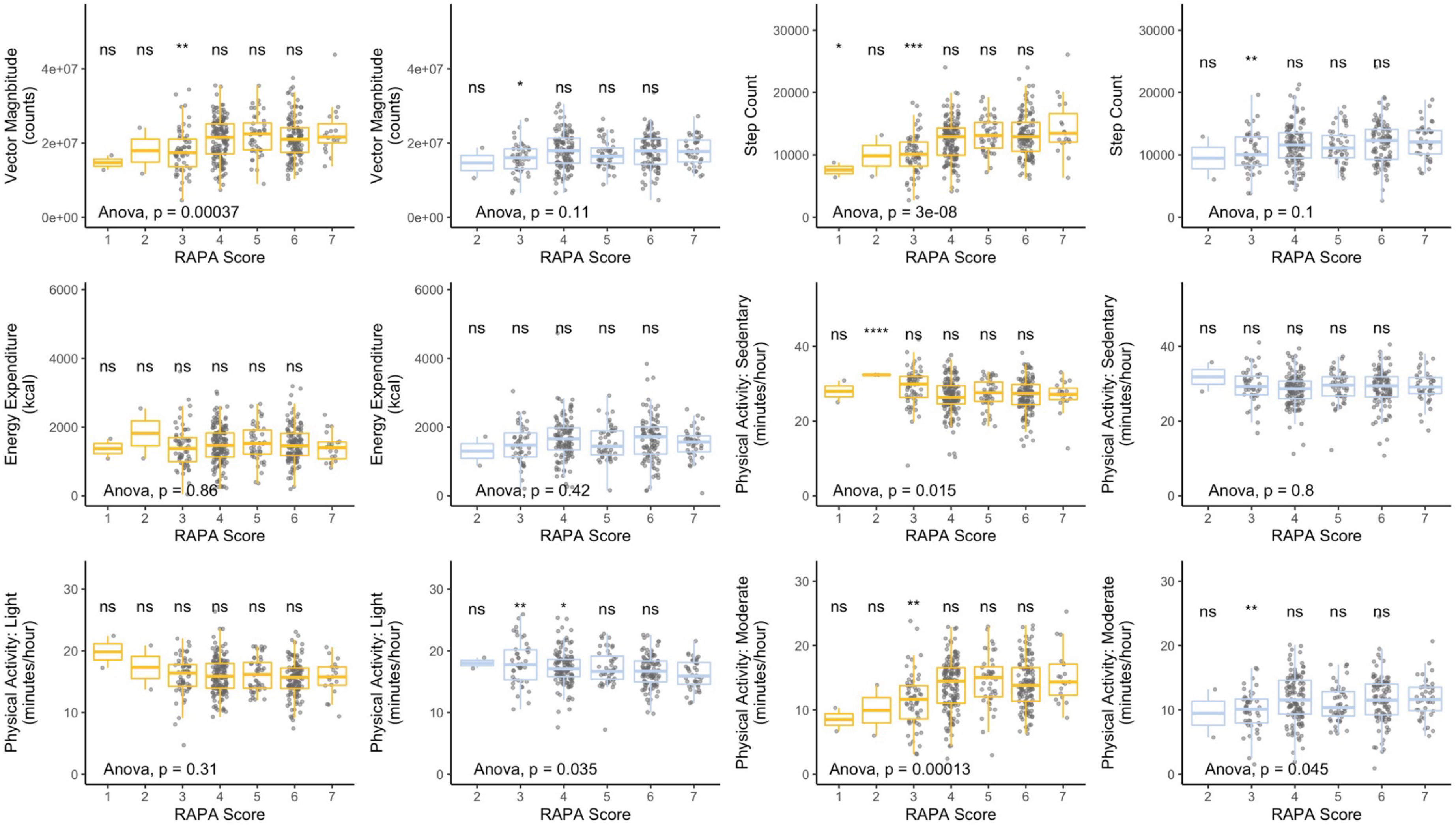


**Supplementary Figure 4: Results in activity variables stratified by self-rated physical activity (assessed via RAPA questionnaire) and sex (women = yellow, men = blue).** Statistical significance of difference between means was assessed by ANOVA. Difference between the highest self-reported activity (RAPA = 7) and the other RAPA groups were tested for statistical significance with t-test. In contrast to results in Supplementary Table 1, Physical Activity is presented in minutes/hour. It can be transformed as follows: [minutes/hour]= ([hours/day]*60)/24.

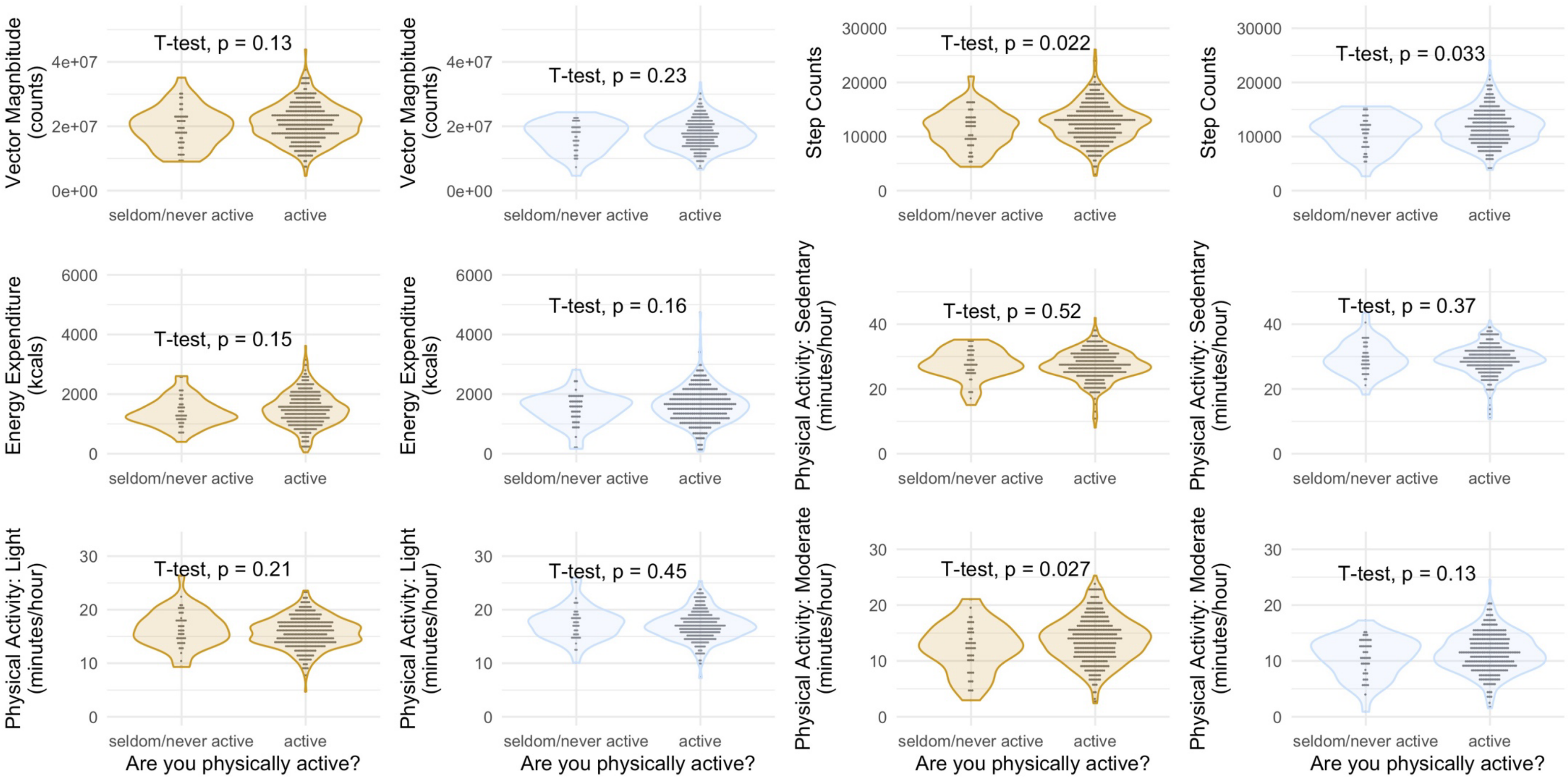


**Supplementary Figure 5: Sex-stratified violin plots of accelerometric activity variables of participants that reported to be seldom/never active or to be active (women = yellow, men = blue).** Statistical significance of differences was assessed by t-test. In contrast to results in Supplementary Table 1, Physical Activity is presented in minutes/hour. It can be transformed as follows: [minutes/hour]= ([hours/day]*60)/24.

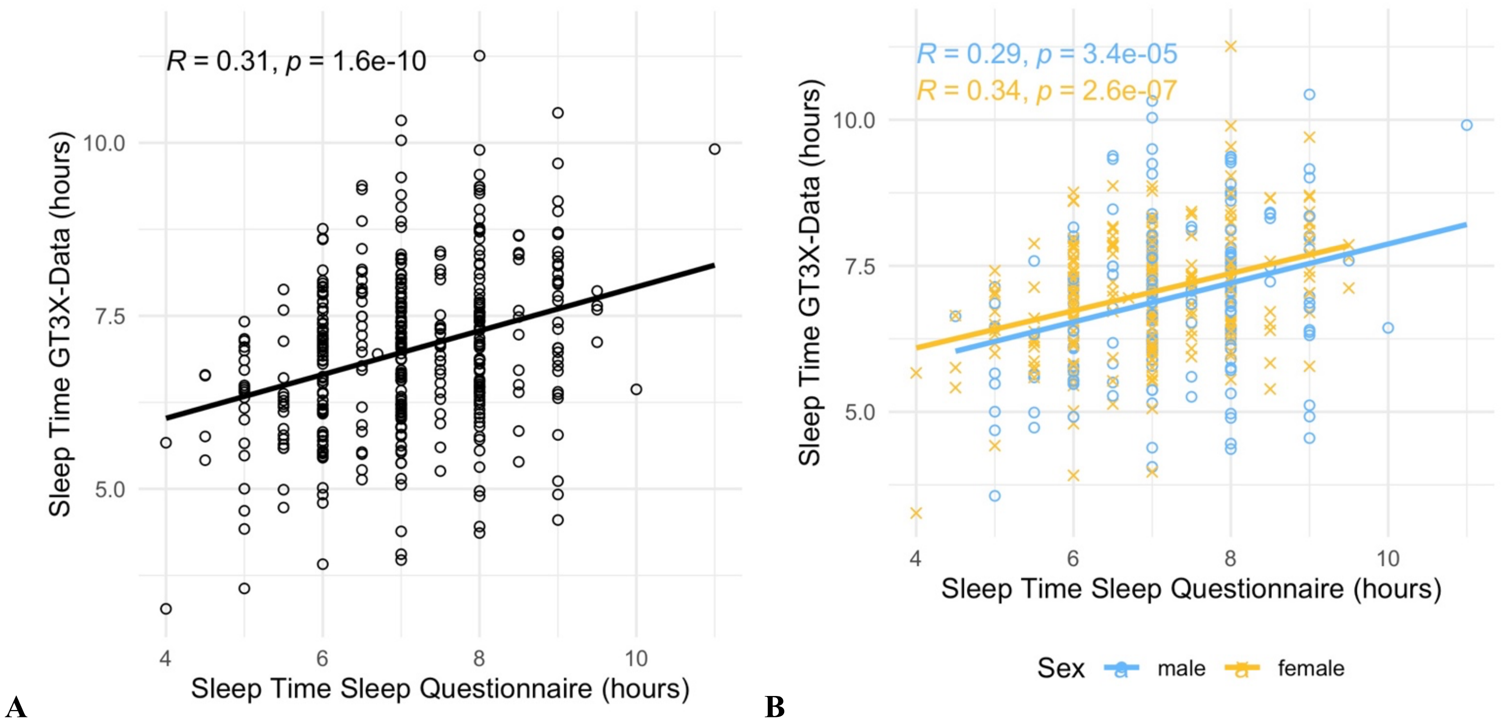


**Supplementary Figure 6: Scatterplots of sleep time assessed with the GT3X monitor and questionnaire-based sleep time.** A) All participants were included (n=410) and B) sex-stratified subgroup analyses were conducted.

**Supplementary Table 1: Sex-stratified cohort characteristics**

| | | Women | | | | | Men | | | | | |
|---|---|---|---|---|---|---|---|---|---|---|---|---|
| | | n | mean | sd | min | max | n | mean | sd | min | max | p-value |
| Age (years) | | 403 | 75.75 | 3.55 | 67.13 | 94.07 | 357 | 75.46 | 4.08 | 65.95 | 90.03 | 0.295 |
| RAPA Score | 1 | 2 | 0.50 | | | | 0 | 0.00 | | | | 0.012 |
| | 2 | 2 | 0.50 | | | | 2 | 0.56 | | | | |
| | 3 | 59 | 14.64 | | | | 44 | 12.36 | | | | |
| | 4 | 151 | 37.47 | | | | 121 | 33.99 | | | | |
| | 5 | 44 | 10.92 | | | | 47 | 13.20 | | | | |
| | 6 | 126 | 31.27 | | | | 100 | 28.09 | | | | |
| | 7 | 19 | 4.71 | | | | 42 | 11.80 | | | | |
| Seldom/never physically active[1] | yes | 49 | 12.16 | | | | 47 | 13.20 | | | | 0.747 |
| | no | 354 | 87.84 | | | | 309 | 86.80 | | | | |
| Vector Magnitude (counts x1,000) | | 403 | 20,860.00 | 5,793.00 | 4,604.00 | 43,830.00 | 357 | 17,570.00 | 4,516.00 | 4,641.00 | 33,630.00 | <0.001 |
| Step Count | | 403 | 12,430.00 | 3,510.00 | 2,740.00 | 26,070.00 | 357 | 11,620.00 | 3,255.00 | 2,664.00 | 24,040.00 | 0.001 |
| Energy Expenditure (kcal) | | 403 | 1,480.00 | 552.70 | 45.72 | 3,617.00 | 357 | 1,606.00 | 591.60 | 74.54 | 4,734.00 | 0.002 |
| MET | | 403 | 1.42 | 0.16 | 1.03 | 1.91 | 357 | 1.43 | 0.16 | 1.03 | 2.01 | 0.249 |
| Physical Activity: Sedentary (hours/day) [%/hour][a] | | 403 | 8.88 [48] | 1.84 [7] | 3.24 [26] | 16.76 [70] | 357 | 11.6 [50] | 1.84 [7] | 4.28 [28] | 17.44 [73] | <0.001 |
| Physical Activity: Light (hours/day) [%/hour][a] | | 403 | 6.36 [28] | 1.16 [5] | 1.88 [14] | 10.56 [49] | 357 | 6.84 [30] | 1.12 [4] | 2.88 [20] | 10.36 [45] | <0.001 |
| Physical Activity: Moderate (hours/day) [%/hour][a] | | 403 | 5.48 [24] | 1.64 [7] | 0.96 [5] | 10.12 [47] | 357 | 4.56 [20] | 1.4 [6] | 0.36 [2] | 9.8 [42] | <0.001 |
| Average Wear Time (hours/day) | | 403 | 23.09 | 1.35 | 15.59 | 24.00 | 357 | 23.22 | 1.26 | 16.19 | 24.00 | 0.157 |

Note: [a]Please note that the hours per day spent on each activity category do not sum up to the full 24 hours, as the average time per day the device was not worn must be included. The percentage value per hour indicates the share of the respective activity category in all activities recorded per hour (and therefore sums up to 100% as the time during which the device was not worn is not included in the calculation). In addition, the Actilife software package evaluates wear time separately from PA variables by algorithm and wear time sensor, as described in the Methods. Therefore, differences between the sum of PA per day and the average wear time per day are to be expected.

SD: standard deviation, min: minimum, max: maximum, RAPA: Rapid Assessment of Physical Activity, kcal: kilocalorie, MET: Metabolic Equivalent of Task.

**Supplementary Table 2: Correlation table of the whole dataset including imputed values for accelerometric variables. Correlation was assessed as Pearson's r.** Please note that the correlation coefficients for the subgroup with measured accelerometric data may vary from the results shown in the main manuscript because more strict inclusion criteria were applied to the dataset used for analyses that were presented in the main manuscript, as it focusses on the accelerometric data.

| | BMI | | Age | | Cholesterol | | HDL-C | | LDL-C | | Triglycerides | | Glucose | | HbA1c | |
|---|---|---|---|---|---|---|---|---|---|---|---|---|---|---|---|---|
| | r | n | r | n | r | n | r | n | r | n | r | n | r | n | r | n |
| Energy Expenditure (kcals) | 0.39** | 792 | -0.17** | 792 | -0.09* | 792 | -0.13** | 782 | -0.06 | 792 | 0.07 | 792 | 0.08* | 790 | 0.08* | 788 |
| Energy Expenditure (kcals, imputed) | 0.39** | 1098 | -0.16** | 1098 | -0.06 | 1093 | -0.12** | 1078 | -0.03 | 1091 | 0.07* | 1092 | 0.09* | 1090 | 0.09* | 1089 |
| Vector Magnitude (counts) | -0.15** | 792 | -0.15** | 792 | 0.14** | 792 | 0.24** | 782 | 0.08* | 792 | -0.11* | 792 | -0.15** | 790 | -0.07* | 788 |
| Vector Magnitude (counts, imputed) | -0.15** | 1098 | -0.14** | 1098 | 0.17** | 1093 | 0.22** | 1078 | 0.12** | 1091 | -0.07* | 1092 | -0.14** | 1090 | -0.06* | 1089 |
| Step Count | -0.25** | 792 | -0.14** | 792 | 0.1* | 792 | 0.2** | 782 | 0.07* | 792 | -0.14** | 792 | -0.17** | 790 | -0.11* | 788 |
| Step Count (imputed) | -0.25** | 1098 | -0.13** | 1098 | 0.13** | 1093 | 0.18** | 1078 | 0.1* | 1091 | -0.1* | 1092 | -0.17** | 1090 | -0.1** | 1089 |
| Physical activity: Sedentary | 0.06 | 792 | 0.09 | 792 | -0.01 | 792 | -0.11* | 782 | 0.02 | 792 | 0.01 | 792 | 0.04 | 790 | -0.01 | 788 |
| Physical activity: Sedentary (imputed) | 0.04 | 1098 | 0.11** | 1098 | -0.01 | 1093 | -0.11** | 1078 | 0.02 | 1091 | 0.02 | 1092 | 0.03 | 1090 | 0.02 | 1089 |
| Physical activity: Light | 0.13** | 792 | 0.05 | 792 | -0.07* | 792 | -0.14** | 782 | -0.04 | 792 | 0.02 | 792 | 0.07* | 790 | 0.08* | 788 |
| Physical activity: Light (imputed) | 0.12** | 1098 | 0.07* | 1098 | -0.06* | 1093 | -0.14** | 1078 | -0.03 | 1091 | 0.03 | 1092 | 0.07* | 1090 | 0.09* | 1089 |
| Physical activity: Moderate | -0.17** | 792 | -0.14** | 792 | 0.12* | 792 | 0.24** | 782 | 0.06 | 792 | -0.11* | 792 | -0.16** | 790 | -0.09* | 788 |
| Physical activity: Moderate (imputed) | -0.16** | 1098 | -0.13** | 1098 | 0.16** | 1093 | 0.22** | 1078 | 0.11** | 1091 | -0.08* | 1092 | -0.16** | 1090 | -0.08* | 1089 |

Note: BMI: Body Mass Index, HDL-C: high density lipoprotein cholesterol, LDL-C: low density lipoprotein cholesterol, HbA1C: hemoglobin A1c, kcal: kilocalorie. *p<0.05; **p<0.001
